# The Association Study Between Cytokines and the Risk for Cerebral Palsy

**DOI:** 10.1155/mi/3742331

**Published:** 2025-03-16

**Authors:** Baotian Wang, Fan Wang, Li Yang, Junhong Jiang, Jiulai Tang, De Wu

**Affiliations:** ^1^Department of Pediatrics, The First Affiliated Hospital of Anhui Medical University, Hefei 230022, Anhui, China; ^2^Department of Outpatient, The First Affiliated Hospital of Anhui Medical University, Hefei 230022, Anhui, China

**Keywords:** biomarker, cerebral palsy, NSE, sIL2-R*α*, TNF-*α*

## Abstract

**Background:** Cerebral palsy (CP) is a debilitating condition characterized by abnormal movement or posture beginning early in development. Recent evidence has shown that immunological abnormalities are associated with an increased risk of CP. However, there are no valuable biomarkers for CP diagnosis.

**Methods:** In this case–control study, we recruited 108 children with CP and 52 healthy children as controls. The white blood cell (WBC) counts and the levels of inflammatory markers (interleukin-1*β* (IL-1*β*), sIL-2R, interleukin-6 (IL-6), IL-8, IL-10, and tumor necrosis factor-*α* (TNF-*α*)), neuron-specific enolase (NSE), immunoglobulin E (IgE), and C3/C4 in the blood were measured and the results were statistically analyzed. Subgroup analyzes based on age, complications, and clinical subtypes were also carried out.

**Results:** Compared with the controls, CP patients had elevated levels of NSE, sIL-2R, and TNF-*α*. There were no differences in WBC count, IL-1*β*, IL-6, IL-8, IL-10, IgE, C3, or C4. Subgroup analysis revealed significant differences in the personal–social developmental quotient (DQ) among the different CP subtypes. We found that TNF-*α*, sIL-2R, gross motor DQ, and adaptive DQ were greater in children with CP without epilepsy (EP) than in those with EP. Correlation analysis revealed positive correlations between TNF-*α* and sIL-2R, gross motor DQ, fine motor DQ, adaptive DQ, and personal–social DQ; moreover, sIL-2R was positively correlated with TNF-*α*, gross motor DQ, adaptive DQ, personal–social DQ, and eosinophil (EO) count and negatively correlated with age. NSE and TNF-*α* were associated with a 1.64-fold and 1.66-fold increased risk of CP, respectively. The peripheral blood NSE and TNF-*α* levels exhibited good diagnostic value for CP. Moreover, receiver operating characteristic (ROC) curve analysis revealed a significant increase in the area under the curve (AUC) when these indicators were combined.

**Conclusions:** This study revealed significant associations between NSE and TNF-*α* and CP risk, suggesting that NSE and TNF-*α* might be useful blood biomarkers for identifying patients at high risk of CP.

## 1. Introduction

The clinical neurological illness known as cerebral palsy (CP) is defined by a variety of symptoms, including abnormal posture or movement, and it usually manifests early in life [1–4]. CP is caused by abnormalities in the fetal or infant brain and results in activity restriction or disability [1]. Cognitive, sensory, language, behavior, musculoskeletal disorders, and epilepsy (EP) can all coexist with motor disability. The etiology of CP is complex, multifaceted, and poorly understood, with numerous factors potentially contributing to its development [4]. The diagnosis of CP tends to be complicated and delayed and typically occurs at 1–2 years of age or older. Thus, early identification of CP is critical, since it allows for treatments that can have a major influence on children's health outcomes.

A prevalent mechanism for causing brain damage has been proposed to be an aberrant cytokine response, and inflammation is thought to be a significant causative element of negative neurological consequences [5]. Cytokines are immune system proteins that are released in response to infection or injury [6]. They are essential for the immune response and can either protect the body from infection or cause damage to tissues. Elevated concentrations of proinflammatory cytokines, including interleukin-1*β* (IL-1*β*) [7–10], interleukin-6 (IL-6) [5, 11–13], tumor necrosis factor-*α* (TNF-*α*) [14–22], and other cytokines [23–26], are present in the blood and brain tissue of individuals with CP. Other studies, however, have demonstrated that there is no statistically significant correlation between cytokine levels and CP [27–30]. This discrepancy raises the following question: do the levels of cytokines in the blood of children with CP increase due to CP itself or due to other factors (such as allergies)? A biomarker that could be used to predict the likelihood of developing CP would be extremely useful for clinical application and to aid in early diagnosis. Understanding the function of cytokines in the pathogenesis of this disease may reveal novel therapeutic targets and strategies for preventing or treating CP.

In this study, we analyzed white blood cell (WBC) counts and inflammatory marker (TNF-*α*, IL-1*β*, sIL-2R, IL-6, IL-8, and IL-10), neuron-specific enolase (NSE), immunoglobulin E (IgE), and complement factor C3/C4 levels in the blood of children with CP and healthy peers to identify a sensitive and simple serum biological marker that could be a potential target for CP diagnosis and treatment.

## 2. Methods

### 2.1. Study Design

The First Affiliated Hospital of Anhui Medical University served as the site of this case–control investigation. Between January 2016 and December 2023, 108 children with CP and 52 healthy controls were recurited from our institution. Our institution's Ethical Committee for Clinical Research approved this study (No. PJ20230232). All parents of both healthy and CP patients provided written informed consent for the collection of blood samples and the publication of the results. CP was diagnosed using the definition, classification, and diagnostic criteria recommended by the Chinese guidelines for CP diagnosis and treatment (2022) [31]. A total of 41 female and 67 male CP patients between the ages of 6 months and 16 years were included. Blood samples were collected by skilled doctors on the second day of patient's stay. The following criteria were used to exclude patients: 1) had fever, cough, or respiratory disease for 2 months or 2) had a history of anti-inflammatory or other drug use, botulinum toxin injections, surgery or brain stimulation, congenital malformations, metabolic disorders, or other serious pediatric illnesses that could increase the concentrations of TNF-*α*, sIL-2R, IL-1*β*, IL-6, IL-10, IL-8, and NSE. The development of the children was evaluated using the Gesell Developmental Schedules (GDS). The GDS is a screening examination used to identify developmental quotients (DQs) in language, adaptive behavior, fine motor skills, personal–social behavior, and gross motor skills [32]. Every child with CP had their DQ assessed using the updated GDS. The children with CP were separated into four groups on the basis of their CP subtype [31].

### 2.2. Laboratory Analysis

WBC counts and the ratio of each component cell type were calculated using a Sysmex diagnostic analyzer (Sysmex Corporation, Japan). Using these values, the percentages of neutrophils (*N*), lymphocytes (*L*), and eosinophils (EOs) were calculated. The IgE, C3, and C4 levels were measured via enzyme-linked immunosorbent assay (ELISA) kits (Beckman Colter, CA, USA). TNF-*α*, IL-1*β*, sIL-2R, IL-6, IL-8, and IL-10 levels were measured using ELISA kits (Siemens Healthcare Diagnostics Products Limited, Llanberis, UK) in accordance with the manufacturer's instructions. The minimum detectable concentrations of TNF-*α*, IL-1*β*, sIL-2R, IL-6, IL-8, and IL-10 are 4, 5, 5, 2, 5, and 5 pg/mL, respectively [33]. Following the manufacturer's instructions, the plasma concentration of NSE was measured using an immunoradiometric assay (IRMA) kit (Roche Diagnostics GmbH, Mannheim, Germany).

### 2.3. Statistical Analysis

The mean ± standard deviation (SD) was used to display the data. All of the statistical analyses were carried out with SPSS for Windows version 19.0 (SPSS, Inc., IL, USA). One-way analysis of variance (ANOVA) was used to compare the data between groups, and chi-square tests (*χ*^2^) and *t* tests were used to compare differences in measurement data between groups. Binary logistic regression analysis (odds ratio (OR), 95% confidence interval (CI)) was used as the dependent variable to assess the relationship between each individual marker and CP risk. Candidate markers for the combined receiver operating characteristic (ROC) curves were identified by characterizing the connection between the biomarker and clinical status using the OR obtained from binary logistic regression analysis. A logistic regression model was used to generate ROC curves. The general degree of discrimination was evaluated according to the area under the curve (AUC). Pearson correlation analysis was performed for the indicated variables. Additionally, GraphPad Prism version 6.0 (GraphPad Software, CA, USA) was used to plot the data. A *p* value of less than 0.05 indicated a statistically significant difference.

## 3. Results

The mean age of the 67 male and 41 female patients in the CP group was 3.35 ± 2.79 years. The 52 healthy children in the control group, including 30 boys and 22 girls, had a mean age of 3.5 ± 2.36 years. A total of 108 data points were obtained for NSE, inflammatory marker, and WBC counts; 103 data points were obtained for IgE, C3, and C4; and 89 data points were obtained for DQs.  Table 1 displays all the participants' clinical characteristics. There were no significant differences in sex or age distribution between the CP group and the control group (Table 1).

### 3.1. Overall Analysis

In our study, the concentrations of IL-1*β*, sIL-2R, IL-6, IL-10, IL-8, NSE, TNF-*α*, IgE, C3, and C4 were measured in the plasma, and the WBC count was measured in the serum.  Table 2 displays the primary data as the means ± SDs. According to the statistical analysis, the children with CP had significantly higher levels of sIL-2R, TNF-*α*, and NSE than the controls did (*p*  < 0.01; Table 2). There was no significant difference between the CP and control groups in terms of WBC, *N*, *L*, and EO counts or IgE, C3, and C4 levels (*p*  > 0.05; Table 2). The concentrations of IL-6, IL-1*β*, IL-8, and IL-10 in most participants were below the lower limit of detection and the differences were not statistically significant.

### 3.2. Subgroup Analysis

NSE, sIL-2R, and TNF-*α* levels and DQ scores were compared among the different subtypes of CP (Table 3). We found developmental delay in all functional domains among children with CP. The personal-social DQ of the various CP subtypes varied significantly (*p*  < 0.05; Table 3). However, the groups did not differ significantly in terms of TNF-*α*, sIL-2R, NSE, or other DQ scores (*p*  > 0.05; Table 3).

To determine whether EP affected the results, we divided the children with CP into two groups for statistical analysis. We found that children with CP without EP had significantly greater TNF-*α*, NSE, and sIL-2R levels and DQ scores than did those with EP, but the differences in sIL-2R and TNF-*α* levels and gross motor and adaptive DQ scores were statistically significant (*p*  < 0.05; Table 4).

Since children with CP underwent rehabilitation training prior to being included in this research, we divided them into two groups for comparative analysis to study whether rehabilitation treatment had an impact on blood parameters. Only the NSE and sIL-2R levels were significantly different (*p*  < 0.05; Table 5); however, the plasma levels of TNF-*α*, sIL-2R, and NSE were significantly greater in the nonrehabilitation group than in the rehabilitation group.

### 3.3. Correlation Analysis

The correlations between blood parameters and age and DQ were evaluated.  Table 6 displays the Pearson correlation coefficients. Correlation analysis revealed a positive relationship between sIL-2R levels and TNF-*α* levels in the CP group (*r* = 0.36, 95% CI: 0.21–0.55, *p*=0.01). The GDS–DQ scores and the sIL-2R and TNF-*α* levels in the CP group were positively correlated (*p*  < 0.05; Table 6). In the CP group, we observed a negative association between sIL-2R and age (*r* = −0.51, 95% CI: −0.66 to −0.41, *p* ≤ 0.01), but a positive correlation between sIL-2R and EO count (*r* = 0.35, 95% CI: 0.07–0.66, *p*=0.01). NSE levels and other variables in the CP group, however, did not correlate (*p*  > 0.05; Table 6).

### 3.4. Logistic Regression Analysis

The effects of different variables on CP risk were investigated using multivariate binary logistic regression analysis and the “enter” variable selection strategy and the findings are presented in Table 7. We found that sIL-2R, TNF-*α*, and NSE were risk factors for CP (*p*  < 0.05; Table 7). After adjustments for sIL-2R, TNF-*α*, and NSE were made, NSE and TNF-*α* were found to contribute to a 1.65-fold greater risk of CP (OR: 1.65, 95% CI: 1.28–2.12, *p* ≤ 0.01 and OR: 1.65, 95% CI: 1.23–2.21, *p* ≤ 0.01, respectively), whereas sIL-2R was not associated with CP (OR: 1.001, 95% CI: 0.997–1.006, *p*=0.57). After adjustments for TNF-*α* and NSE were made, we found that NSE and TNF-*α* were linked to 1.64-fold (95% CI: 1.29–2.09, *p* ≤ 0.01) and 1.66-fold (95% CI: 1.25–2.21, *p* ≤ 0.01) increases in CP risk, respectively.

**Figure 1:**
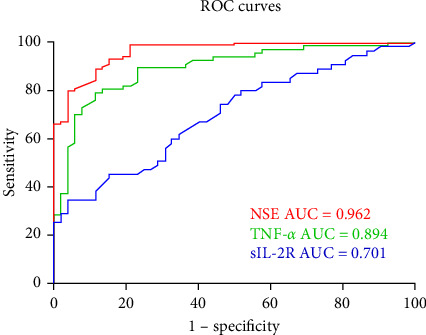
Receiver operating characteristic (ROC) predicted cerebral palsy (CP) based on the levels of neuron-specific enolase (NSE), sIL-2R, and tumor necrosis factor-*α* (TNF-*α*).

**Figure 2:**
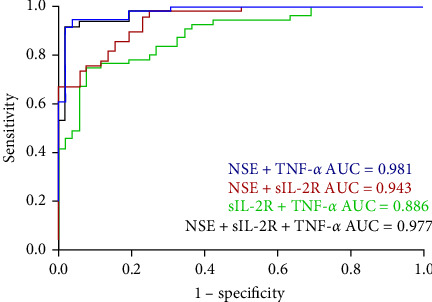
Receiver operating characteristic (ROC) curve for predicting cerebral palsy (CP) based on combine.

The effectiveness of these biomarkers in diagnosing CP was then evaluated using ROC curve analysis.  Figure 1 shows the AUCs of the three parameters measured individually. The ROC curves demonstrated the relatively substantial diagnostic significance of NSE (AUC = 0.962, 95% CI: 0.936–0.989, *p* ≤ 0.001), TNF-*α* (AUC = 0.894, 95% CI: 0.836–0.952, *p* ≤ 0.001) and sIL-2R (AUC = 0.701, 95% CI: 0.603–0.799, *p* ≤ 0.001).  Figure 2 shows the combined ROC curves generated via logistic regression analysis. Analysis of the ROC curves revealed that the combination of sIL-2R + TNF-*α* (AUC = 0.886,95% CI: 0.825–0.947, *p* ≤ 0.001), sIL-2R + NSE (AUC = 0.943,95% CI: 0.904–0.983, *p* ≤ 0.001), TNF-*α* + NSE + sIL-2R (AUC = 0.977,95% CI: 0.953–1.000, *p* ≤ 0.001) and TNF-*α* + NSE (AUC = 0.981,95% CI: 0.961–1.000, *p* ≤ 0.001) had significant diagnostic value. As illustrated in Figure 2, the AUC of the combined ROC curves was greater than that of the individual ROC curves.

## 4. Discussion

In our study, we found that TNF-*α*, NSE, and sIL-2R levels were increased in the blood of children with CP and CP risk was significantly correlated with both NSE and TNF-*α*, which might be important for the early identification of CP in young children who have not been diagnosed.

The current definition of CP states that it is a group of postural and movement impairments that limit activity and are caused by nonprogressive abnormalities in the developing fetal or neonatal brain [34]. However, we believe that CP is a varied mix of etiological diseases that start early in development and are characterized by aberrant movement or posture rather than a single disease entity [2–4]. Injury can last for several months or years, in addition to the developmental disturbance caused by initial trauma to the developing brain [35]. Although the cause and pathophysiology of CP are poorly understood, an increasing number of studies have demonstrated a link between cytokines and CP risk [7, 12, 14, 21, 23–26]. Analyzing the molecular composition of blood, particularly cytokines, can provide insights into the pathophysiology of CP. Elevated levels of pro-inflammatory cytokines may lead to brain damage and CP through several mechanisms. First, cytokines have the ability to break down the blood–brain barrier, which allows dangerous chemicals to enter the brain. Second, cytokines can cause brain cell damage by inducing the generation of reactive oxygen compounds. In contrast to earlier studies [5–13, 24], our study revealed that children with CP had considerably higher levels of TNF-*α* and sIL-2R than healthy controls did, but that the levels of IL-6, IL-1*β*, IL-8, and IL-10 were not significantly different. After further analysis, we discovered a strong correlation between TNF-*α* and CP incidence. According to the ROC analysis, AUCs greater than 0.7 and *p* values less than 0.001 suggested that sIL-2R and TNF-*α* might be used as biomarkers for the diagnosis of CP. Moreover, the ROC curve of the combined model revealed that the combined model had greater diagnostic value than either of the independent diagnostic factors alone (Figure 1), as shown in Figure 2. Moreover, NSE was strongly associated with CP and was significantly greater in children with CP than in healthy controls. Furthermore, NSE, sIL-2R, and TNF-*α* were found to be risk factors in isolated binary logistic analyses; however, only NSE and TNF-*α* were found to be risk factors in the combined analysis of the three variables. Thus, the highest diagnostic value was found for the combined ROC curve of TNF-*α* and NSE (Figure 2). The adoption of combination models, evaluated via ROC curves, can increase the value of diagnosing each particular biomarker and improve the understanding of connections as tools to advance our knowledge of the etiological mechanisms of CP. These models can also predict the functional status and possible complications of children with CP to provide better guidance for clinical treatment and therefore enhance the standard of living for children with CP.

The NSE level is a marker of neuronal damage after neurodegenerative disease [36]. Hyperbilirubinemia and lipemia have no effect on the evaluation of NSE because it is soluble and stable in biological fluids [37]. Our study revealed that NSE is elevated in children with CP and that a high NSE level is linked to an increase in CP risk of 1.64-fold (95% CI: 1.29–2.09, *p* ≤ 0.01). NSE reflects the severity of brain injury and is expected to be correlated with DQ; however, correlation analysis revealed no correlation between NSE and DQ, while both sIL-2R and TNF-*α* were correlated with some DQ scores (Table 6), indicating that sIL-2R and TNF-*α* may be related to the pathogenesis of CP.

Since elevated sIL-2R levels are believed to indicate ongoing illness progression in patients with autoimmune illnesses, sIL-2R participates in the etiology of these diseases through effector cell transactivation [38]. As in our earlier research [39], in the present study, sIL-2R levels exhibited a lower fold change between CP patients and healthy controls than did TNF-*α* and NSE levels. NSE also had the lowest diagnostic value (AUC of 0.701) compared with TNF-*α* and NSE (AUC of 0.962 and 0.894, respectively). Although multivariate regression analysis revealed that sIL-2R was not associated with CP, regression analysis revealed that the concentration of sIL-2R was greater in children with CP than in healthy controls. However, multivariate regression analysis revealed that sIL-2R was not associated with CP, with an OR of 1.001 (95% CI: 0.997–1.006, *p*=0.57). To investigate whether the increase in sIL-2R was associated with other factors, we analyzed whether sIL-2R was positively correlated with EO count and TNF-*α*. These results suggested that an increase in sIL-2R may be associated with TNF-*α* levels and/or allergies. Although several studies have suggested a correlation between sIL-2R and CP [26], our research indicated no such correlation. The variations in outcomes may be associated with the samples included in the study.

TNF-*α* is an essential proinflammatory cytokine and is crucial for systemic inflammation, apoptosis, and necrosis [40]. In our study, we discovered that children with CP had higher levels of TNF-*α*. Currently, it is difficult to determine whether increased TNF-*α* causes CP or whether CP causes increased TNF-*α*. We found positive correlations between TNF-*α* and adaptive DQ, personal–social DQ, gross motor DQ, and fine motor DQ. This finding is very interesting because it implies that TNF-*α* may protect children with CP, which may indicate that CP causes an increase in TNF-*α*. We also found that TNF-*α* was linked to an increase in CP risk of 1.66-fold (95% CI: 1.25–2.21, *p* ≤ 0.01), and the effect of NSE was less pronounced. The ROC curve indicated that TNF-*α* (AUC = 0.894, 95% CI: 0.836–0.952, *p* ≤ 0.001) has significant diagnostic value. This finding may imply that elevated TNF-*α* causes CP and indicates a persistent inflammatory response in children with CP. Although some studies that suggest that there is no link between TNF-*α* and CP [27–30, 41], on the basis of our research, we believe that there is a significant association between TNF-*α* and CP and that TNF-*α* might be a useful blood biomarker for CP. sIL-2R is likely a less valuable marker because sIL-2R levels are increased only in some children with CP, wheras TNF-*α* levels are increased in almost all children with CP.

The different types of CP are related to different types of brain injury, the pathogenesis of each type is unclear and the clinical manifestations differ. To better evaluate the development of CP in children, we used the GDS rather than the Gross Motor Function Classification System (GMFCS). Our research revealed that DQ scores were poor in individuals with different types of CP (Table 3). Although some studies [42–44] have suggested that epileptic seizures cause an increase in cytokines, we detected reduced cytokine levels (Table 4), although only the differences in sIL-2R and TNF-*α* levels were statistically significant (*p*  < 0.05; Table 4). Previous research has shown that rehabilitation interventions can reduce the concentration of TNF-*α* [43], but our research revealed no statistically significant change in TNF-*α* levels in patients with or without rehabilitation interventions, which may be related to the samples we collected (Table 5).

Overall, the results indicated that the levels of TNF-*α*, sIL-2R, and NSE were greater in children with CP than in healthy peers and that there was a significant correlation between TNF-*α* and NSE and CP, suggesting that NSE and TNF-*α* might be useful blood biomarkers for identifying children who are at greater risk for CP. However, additional research is required to determine the exact underlying mechanism involved and to identify potential targets for treatment or prevention.

## Figures and Tables

**Table 1:** Clinical characteristics of all participants.

Characteristic	CP (*n* = 108)	Controls (*n* = 52)
Gender (M:F)	67:41	30:22
Age (years) <1 1 ≤ years < 3 3 ≤ years < 10 ≥10	9 45 48 6	0 25 26 1
Type of CP Spast Hemiplega Dyskinetic Mixed Other	46 33 11 12 6	N/A
CP with EP (Y:N)	17:91	N/A
Rehabilitative treatment (Y:N)	71:37	N/A

Abbreviations: CP, cerebral palsy; EP, epilepsy.

**Table 2:** Laboratory characteristics of CP and healthy controls (*x* ± *s*).

Group	NSE	TNF-*α*	sIL-2R	WBC	*N*	*L*	EO	IgE	C3	C4
CP	25.54 ± 15.58	13.27 ± 4.31	750.31 ± 369.62	7.99 ± 2.54	32.67 ± 12.21	57.87 ± 12.46	0.23 ± 0.15	150.38 ± 196.93	1.16 ± 0.19	0.26 ± 0.08
Con	9.21 ± 4.32	7.35 ± 2.26	526.54 ± 166.47	8.12 ± 2.43	37.48 ± 20.41	54.73 ± 20.34	0.22 ± 0.18	164.62 ± 166.2	1.2 ± 0.22	0.27 ± 0.12
*t*	7.40	9.65	4.07	0.27	1.47	0.96	0.25	0.33	1.01	0.29
*p*	0.01	0.01	0.01	0.79	0.15	0.34	0.80	0.74	0.32	0.78

Abbreviations: CP, cerebral palsy; EO, eosinophil; IgE, immunoglobulin E; NSE, neuron-specific enolase; TNF-*α*, tumor necrosis factor-*α*; WBC, white blood cell.

**Table 3:** Comparison between different types of CP.

Group	NSE	TNF-*α*	sIL-2R	Gross motor DQ	Fine motor DQ	Adaptive DQ	Language DQ	Personal–social DQ
Spast	24.68 ± 16.46	13.3 ± 4.2	743.98 ± 386.61	50.06 ± 29.35	46.89 ± 27.7	54.11 ± 28.1	55 ± 24.45	53.83 ± 24.08
Dyskinetic	28.63 ± 8.62	11.39 ± 3.86	682 ± 222.03	18.4 ± 12.7	25.4 ± 21.732	30.2 ± 19.73	29 ± 14.78	29.6 ± 20.64
Mixed	30.6 ± 18.25	13.59 ± 5.96	748.83 ± 295.63	24.33 ± 2.89	22.67 ± 6.66	21.33 ± 2.52	28 ± 13	21.33 ± 2.52
Other	22.61 ± 5.33	14.33 ± 2.94	891.67 ± 443.4	48.5 ± 30.41	40 ± 19.8	48 ± 31.11	32.5 ± 14.85	31 ± 9.9
*F*	0.62	0.32	0.17	2.45	1.46	2.14	2.92	3.19
*p*	0.6	0.81	0.92	0.09	0.25	0.12	0.05	0.04

Abbreviations: CP, cerebral palsy; DQ, developmental quotient; NSE, neuron-specific enolase; TNF-*α*, tumor necrosis factor-*α*.

**Table 4:** Comparison between with EP and without EP.

Group	NSE	TNF-*α*	sIL-2R	Gross motor DQ	Fine motor DQ	Adaptive DQ	Language DQ	Personal–social DQ
CP with EP	18.6 ± 7.07	11.02 ± 3.34	626.14 ± 175.77	23.5 ± 7.9	29.75 ± 16.11	25.75 ± 15.2	35 ± 13.34	35.5 ± 6.4
CP without EP	26.86 ± 16.42	13.87 ± 4.37	792.71 ± 408.85	44.54 ± 29.11	41.67 ± 27.16	49.25 ± 27.7	47.67 ± 25.33	45.88 ± 26.23
*t*	1.97	2.27	2.1	2.95	0.84	2.48	0.97	1.66
*p*	0.05	0.03	0.04	0.01	0.41	0.04	0.34	0.11

Abbreviations: CP, cerebral palsy; DQ, developmental quotient; EP, epilepsy; NSE, neuron-specific enolase; TNF-*α*, tumor necrosis factor-*α*.

**Table 5:** Comparison of rehabilitation treatment and no rehabilitation treatment.

Group	NSE	TNF-*α*	sIL-2R
untreatment	30.73 ± 15.23	14.69 ± 4.14	1048 ± 537.41
treatment	21.59 ± 15.25	12.53 ± 4.26	675.89 ± 275.8
*t*	2.15	2	2.22
*p*	0.04	0.05	0.04

Abbreviations: NSE, neuron-specific enolase; TNF-*α*, tumor necrosis factor-*α*.

**Table 6:** Correlation analysis of CP group.

Biomakers	NSE	TNF-*α*	sIL-2R	Gross motor DQ	Fine motor DQ	Adaptive DQ	Language DQ	Personal–social DQ	WBC	*N*	*L*	EO	IgE	C3	C4	Age
NSE	*r* = 1	—	—	—	—	—	—	—	—	—	—	—	—	—	—	—
TNF-*α*	—	*r* = 1	*r* = 0.36, 95% CI: 0.21–0.55, *p*=0.01	*r* = 0.54, 95% CI: 0.14–0.8, *p*=0.03	*r* = 0.55, 95% CI: 0.2–0.81, *p*=0.03	*r* = 0.77, 95% CI: 0.5–0.93, *p*=0.01	—	*r* = 0.61, 95% CI: 0.22–0.91, *p*=0.01	—	—	—	—	—	—	—	—
sIL-2R	—	*r* = 0.36, 95% CI: 0.21–0.55, *p*=0.01	*r* = 1	*r* = 0.58, 95% CI: −0.03 to 0.85, *p*=0.03	—	*r* = 0.59, 95% CI: 0.15–0.84, *p*=0.03	—	*r* = 0.6, 95% CI: 0.25–0.83, *p*=0.02	—	—	—	*r* = 0.35, 95% CI: 0.07–0.66, *p*=0.01	—	—	—	*r* = −0.51, 95% CI: −0.66 to −0.41, *p* ≤ 0.01

Abbreviations: CI, confidence interval; CP, cerebral palsy; DQ, developmental quotient; EO, eosinophil; IgE, immunoglobulin E; NSE, neuron-specific enolase; TNF-*α*, tumor necrosis factor-*α*; WBC, white blood cell.

**Table 7:** Coeffects of risk factors on CP.

Biomakers	*B*	S.E	*p*	OR	95% CI for OR	
					Lower	Upper
NSE	0.56	0.1	0.01	1.75	1.43	2.14
TNF-*α*	0.54	0.1	0.01	1.71	1.42	2.06
sIL-2R	0.004	0.001	0.01	1.004	1.002	1.006

Abbreviations: CI, confidence interval; CP, cerebral palsy; NSE, neuron-specific enolase; OR, odds ratio; TNF-*α*, tumor necrosis factor-*α*.

## Data Availability

All data generated or analyzed during this study are included in this published article.
